# Cor Triatriatum Sinister Identified after New Onset Atrial Fibrillation in an Elderly Man

**DOI:** 10.1155/2014/674018

**Published:** 2014-12-29

**Authors:** Ignacio A. Zepeda, Peter Morcos, Luis R. Castellanos

**Affiliations:** ^1^Department of Medicine, University of California San Diego, La Jolla, CA 92161, USA; ^2^Division of Cardiovascular Medicine and Sulpizio Family Cardiovascular Center, University of California San Diego, 9434 Medical Center Drive, La Jolla, CA 92037, USA

## Abstract

A 73-year-old man with new onset atrial fibrillation with rapid ventricular response underwent transthoracic echocardiography that revealed an echogenic linear structure along the left atrium, suggestive of cor triatriatum sinister (CTS). CTS was confirmed with transesophageal echocardiography which demonstrated a proximal accessory atrium receiving pulmonary venous flow separated from a distal true atrium by a fibromuscular membrane with a large fenestration allowing flow between the chambers. In CTS, the left atrium is divided into proximal and distal chambers by a fenestrated fibromuscular septum. This cardiac anomaly accounts for 0.1% of cases of congenital heart disease and rarely presents in adults. CTS is primarily diagnosed with echocardiography and is associated with left atrial enlargement and development of atrial fibrillation. Treatment options depend on size of the communication between proximal and distal chambers, the gradient across the membrane, and the position of pulmonary veins. In some instances, surgical resection of the membrane that divides the left atrium is warranted.

## 1. Introduction 

Cor triatriatum sinister (CTS) is a condition in which the left atrium is divided into two chambers by a fenestrated fibromuscular septum [1]. CTS is generally diagnosed in pediatric populations, with age at presentation depending on the size of the membrane fenestration and the resulting obstruction to inflow [2]. Although there are no unique clinical signs or symptoms that distinguish CTS from other entities, dyspnea has been identified as the most common presenting symptom in adults, with atrial fibrillation and mitral regurgitation as precipitating factors [2]. Echocardiography can diagnose most cases of CTS, measure a gradient between the proximal and distal chambers, and estimate pulmonary artery pressures using Doppler [3]. This rare case report discusses the unique variations in clinical presentation of CTS and the diagnostic challenges a clinician may encounter when presented with this unusual cardiac anomaly.

## 2. Case Report

**Figure 1:**
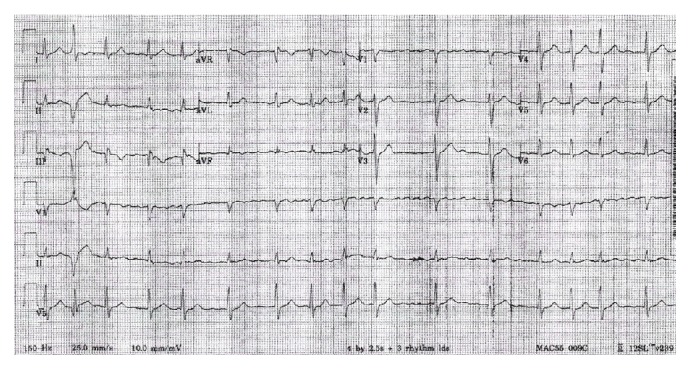
Electrocardiogram shows irregular rhythm consistent with atrial fibrillation with a ventricular rate of 97, no ST segment, or T wave changes suggestive of ischemia.

**Figure 2:**
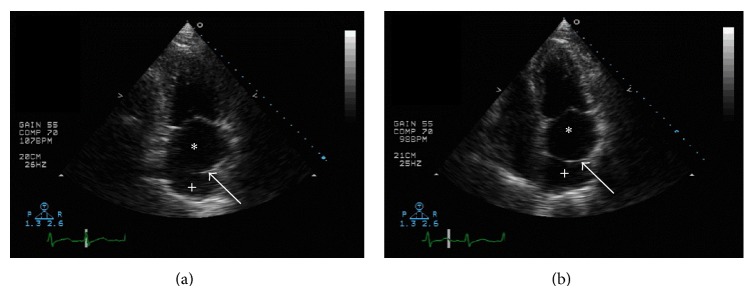
Transthoracic echocardiogram: 2-chamber view (a) and 4-chamber view (b) showing the left atrium divided into proximal (+) and distal (∗) chambers by a fenestrated membrane (arrow).

A 73-year-old man was seen by his primary care provider for routine follow-up of hypertension, hyperlipidemia, esophageal reflux, and generalized anxiety disorder. The patient complained of neuropathic pain in his lower extremities and right hand but denied chest discomfort, dyspnea, lightheadedness, or palpitations. He reported taking aspirin, bisoprolol, bupropion, hydrochlorothiazide, ibuprofen, omeprazole, simvastatin, tamsulosin, and valsartan. Vital signs were blood pressure of 111/69 mmHg, heart rate of 86 bpm, respiratory rate of 20, and oxygen saturation of 96%. On physical exam, he was in no apparent distress but was noted to have an irregularly irregular heart rate and rhythm, no murmurs or rales were detected, and peripheral pulses were strong and symmetrical. An electrocardiogram showed atrial fibrillation (AF) with a ventricular rate of 118 bpm without ST segment or T wave changes suggestive of ischemia (Figure 1). Lab values including complete blood count, thyroid stimulating hormone, basic metabolic panel, liver panel, and fasting lipid panel were unremarkable. The patient was transferred to the emergency department (ED) for further evaluation where he remained stable except for a heart rate varying between 90 and 120 bpm. Rate control was achieved in the ED with several doses of intravenous metoprolol. Given his low risk for complications, the patient was discharged with oral metoprolol and outpatient follow-up. Subsequently, a cardiologist interviewed the patient and ordered a transthoracic echocardiogram (TTE) to evaluate for structural heart disease and a cardiac stress test to evaluate for ischemia as a possible cause for the new onset AF. The cardiac nuclear scan was negative for ischemia. However, the TTE showed normal left ventricular size and systolic function (57% EF by 2D biplane), a severely enlarged left atrium (LA volume index of 57 mL/m^2^), aortic sclerosis without stenosis, mild mitral regurgitation, trace tricuspid regurgitation, and left atrium with an echogenic linear structure suggestive of cor triatriatum (Figure 2). Estimated right ventricular pressure or pulmonary arterial pressure was 35 mmHg. The gradient across the membrane was minimal, estimated at 3 mmHg. A transesophageal echocardiogram (TEE) was obtained in order to further evaluate the possible diagnosis of CTS, evaluate for atrial septal defect (ASD), fenestration of the fibromuscular membrane, identify the location of the pulmonary veins, and distinguish the membrane from a possible supramitral valvular ring.

**Figure 3:**
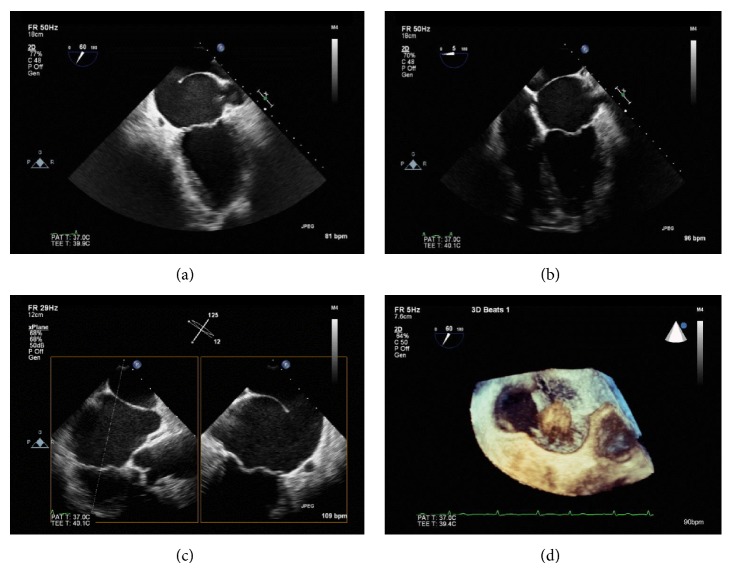
Transesophageal echocardiogram. 2-chamber view (a), 4 chamber view (b), biplane view (c), and 3D mode (d) showing the left atrium divided by a large fenestrated fibromuscular membrane consistent with cor triatriatum sinister.

**Figure 4:**
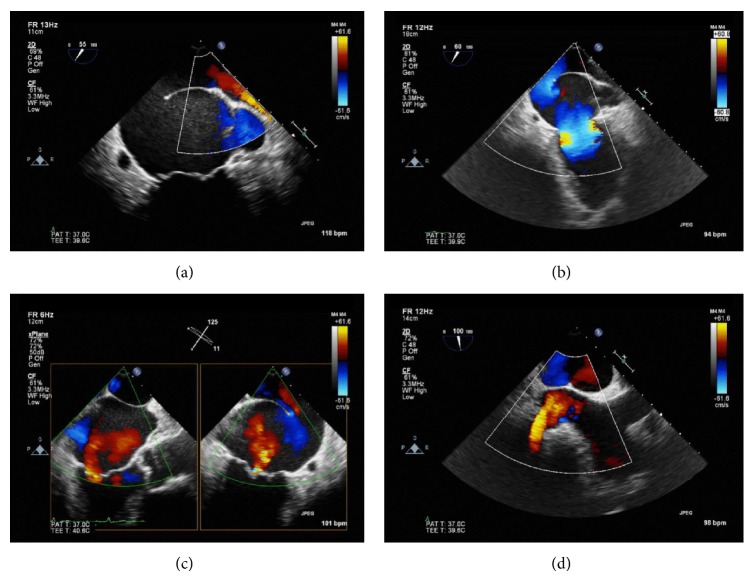
Transesophageal echocardiogram with Doppler color flow analysis. 2-chamber view showing flow from the pulmonary veins into the proximal accessory left atrium (a); flow across the fenestrated membrane into the distal true left atrium and across the mitral valve (b); biplane view showing mitral regurgitation and bidirectional flow across the membrane (c); and bicaval view showing partial attachment of the membrane to the interatrial septum (d).

A TEE with color flow and pulse wave Doppler analysis revealed cor triatriatum with a large communication between the accessory left atrium and the true left atrium (Figure 3). All 4 pulmonary veins entered the accessory left atrium. There was no evidence of atrial septal defect. The left atrial appendage (LAA) was shown to be in the distal chamber, LAA opening measured 1.42 cm in width and 3.1 cm in length, and there was no evidence of thrombi in either the LAA or the left atrium. The mitral valve was structurally normal with central mild regurgitation (Figure 4). The left ventricle was normal in size with preserved systolic function. Given the severely enlarged left atrium, the low-normal left atrium appendage velocities, and a CHA_2_DS_2_-VASc score of 2, the patient was asked to stop taking aspirin and was placed on rivaroxaban for stroke prevention. Given the asymptomatic nature of atrial fibrillation, low gradient across the membrane orifice (nonobstructive), and the patient's unwillingness to undergo further invasive procedures, medical management with rate control was pursued.

## 3. Discussion 

Cor triatriatum sinister is a rare congenital heart malformation that is characterized by separation of the left atrium into a proximal (posterior-superior) accessory chamber that receives inflow from the pulmonary veins and the distal (anterior-inferior) main left atrial chamber by a fenestrated fibromuscular septum [1]. First described by Church in 1868 [4], it accounts for 0.1% to 0.4% of congenital heart defects [5]. CTS is usually diagnosed in children due to early presentation with symptoms of obstruction to pulmonary venous flow and concomitant congenital anomalies, although diagnoses of less severe cases in adult populations have been increasing due to improvements in diagnostic imaging [6].

There are four proposed mechanisms for the embryologic origin of this malformation: (1) an abnormal growth of the septum primum [7], (2) an incomplete incorporation of the embryonic common pulmonary vein into the left atrium [8], (3) the entrapment of the common pulmonary vein by the left horn of the sinus venosus, thereby preventing its incorporation into the left atrium [9], and (4) the persistence of the left superior vena cava that impinges on the developing left atrium [10]. Currently, there is no clear consensus as for the etiology of this rare cardiac defect.

The first and simplest classification scheme for CTS was proposed in 1949 by Loeffler based on the number and size of openings in the intra-atrial membrane [11]. Loeffler classified CTS into three groups: Group I is characterized by complete obstruction from the abnormal membrane and presence of an ASD for drainage of pulmonary venous flow. Group II is characterized by the presence of one or more small openings in the intra-atrial membrane and the possibility of obstruction. A large single opening and little or no obstruction characterize Group III. Other classification schemes take into account the shape of the pulmonary venous chamber (Marin) [12], the site of implantation of the pulmonary veins (Lam) [13], the presence of an ASD in relation to the left atrial chambers (Lupinski) [14], and the presence of anomalous pulmonary venous return and relation of the fossa ovalis with the left atrial chambers (Thilenius) [15].

The majority of pediatric patients with CTS are found to have associated cardiac malformations (77%), most often ASD (53%), anomalous pulmonary venous drainage (28%), and patent ductus arteriosus (18%) [16]. When CTS is diagnosed in adults, it is associated mainly with ASD and mitral regurgitation. Age at presentation is dependent on the size of the membrane fenestration(s) and the presence of associated anomalies. Clinical presentations in previously asymptomatic adults with CTS may be precipitated in part by fibrosis and calcification of the membrane fenestration, leading to narrowing, obstruction, and mimicking of mitral stenosis. This in addition to the increasing incidence of mitral regurgitation with advancing age can contribute to the development of atrial fibrillation [2]. Although the exact mechanism for mitral regurgitation associated with cor triatriatum has not been defined, myxomatous valve degeneration has been implicated [17]. There are no unique clinical features that distinguish CTS from other entities, although dyspnea has been identified as the most common presenting symptom in adults, with palpitations and hemoptysis often added to the clinical picture [2]. On physical exam, a diastolic murmur can be heard, with a loud P2 when pulmonary hypertension is present. The absence of an opening snap or a loud S1 can help differentiate between CTS and mitral stenosis [3].

Although the physiologic consequences of CTS are most similar to mitral stenosis, it has been identified as an important underlying factor in patients presenting with a wide variety of conditions including COPD, atrial fibrillation, cardioembolic stroke, pulmonary artery thrombosis, and esophageal varices [1, 18–20]. Before the widespread use of echocardiography, cases of CTS were commonly first misdiagnosed as mitral valve disease or primary pulmonary hypertension [21]. This highlights the importance of proper diagnostic workup, as clinical presentation can vary and there may be coexisting factors that obscure the underlying cause.

Echocardiography is the mainstay of diagnosis. CTS is first suspected by the appearance of a linear structure in the left atrium in long axis views. An apical four-chamber view will confirm the presence of a definite structure and not an artifact [22]. While TTE can correctly diagnose most cases, it has its limitations in delineating posterior structures in adults and hence may not allow complete characterization of the fibromuscular membrane or the flow across the fenestration in the membrane. Therefore, TEE is used for better visualization of the membrane, fenestrations, measurement of gradients across the membrane, presence of possible ASD, and differentiation from other entities such as supravalvular mitral ring [23]. In this case report, the location of the atrial membrane differentiates CTS from a supravalvular mitral ring. In CTS, the LAA is part of the distal (mitral valve) atrial chamber, whereas the LAA is part of the proximal (pulmonary vein) atrial chamber in patients with a supramitral ring [24]. Additionally, three-dimensional TTE has been used as a less invasive alternative to TEE for the evaluation of CTS [25]. CT and MRI have also been used for the diagnosis of CTS to a lesser extent [26, 27]. However, MRI is limited by being increased in time constraints and may not necessarily provide additional information that can be normally obtained with available echocardiography techniques.

Management of CTS depends on the hemodynamic impact of the atrial membrane. Patients with obstructive symptoms at any age, or those who are asymptomatic but adequate follow-up is not possible, should undergo surgical correction. This is achieved through median sternotomy or right thoracotomy [28]. Because the hemodynamics of obstructive CT mimics the inflow obstruction caused by mitral stenosis, cutoff values reported for mitral stenosis have been used as surrogates for CTS [29]. Saxena et al. reported a 10-year survival of 83% for patients with surgically corrected CTS and NYHA class I or II [30]. Other studies have also shown that the life expectancy of surgically corrected CTS is near to that of the general population [31]. Given the fact that more severe obstructions present at an early age, younger patients are treated with surgical resection more often than older adults in whom a more conservative approach is often implemented. Older patients tend to have less severe obstruction and alternate explanations for symptoms and are at elevated risk for adverse outcomes with surgery. Thus, in older adults, control of concomitant diseases and adequate follow-up alone might be sufficient [2]. Successful balloon dilation of the obstructive membrane has been reported either as a bridge to definitive surgical therapy or as definitive therapy, but long-term outcomes from such strategy remain to be determined [6, 32].

In conclusion, cor triatriatum sinister in the adult is a rare cardiac finding with a wide range of clinical manifestations that may contribute to other cardiac diseases such as mitral regurgitation and atrial fibrillation. TTE is the first line study of choice to assess for presence of CTS. TEE is a valuable diagnostic tool to further assess the classification and severity of the condition as well as guide an appropriate management strategy. Patients who are found to have nonobstructive CTS can be managed medically with standard cardiac risk factor modification. However, routine follow-up with echocardiography is warranted given that, with time, there may be progressive calcification and fibrosis of the membrane that may lead to possible obstruction.

## References

[1] Richardson J. V., Doty D. B., Siewers R. D. (1981). Cor triatriatum (subdivided left atrium). *The Journal of Thoracic and Cardiovascular Surgery*.

[2] Slight R. D., Nzewi O. C., Buell R., Mankad P. S. (2005). Cor-triatriatum sinister presenting in the adult as mitral stenosis: an analysis of factors which may be relevant in late presentation. *Heart, Lung and Circulation*.

[3] Nassar P. N., Hamdan R. H. (2011). Cor Triatriatum sinistrum: classification and imaging modalities. *The European Journal of Cardiovascular Medicine*.

[4] Church W. S. (1868). Congenital malformation of heart: abnormal septum in the left auricle. *Transactions of the Pathological Society of London*.

[5] Niwayma G. (1960). Cor triatriatum. *American Heart Journal*.

[6] Kerkar P., Vora A., Kulkarni H., Narula D., Goyal V., Dalvi B. (1996). Percutaneous balloon dilatation of cor triatriatum sinister. *The American Heart Journal*.

[7] Fowler J. K. (1881). Membranous band in the left auricle. *Transactions of the Pathological Society of London*.

[8] Parsons C. G. (1950). Cor triatriatum. Concerning the nature of an anomalous septum in the left auricle. *British Heart Journal*.

[9] Van Praagh R., Corsini I. (1969). Cor triatriatum: pathologic anatomy and a consideration of morphogenesis based on 13 postmortem cases and a study of normal development of the pulmonary vein and atrial septum in 83 human embryos. *American Heart Journal*.

[10] Gharagozloo F., Bulkley B. H., Hutchins G. M. (1977). A proposed pathogenesis of cor triatriatum: Impingement of the left superior vena cava on the developing left atrium. *American Heart Journal*.

[11] Loeffler E. (1949). Unusual malformation of the left atrium: pulmonary sinus. *Archives of Pathology*.

[12] Marin Garcia J., Tandon R., Lucas R. V., Edwards J. E. (1975). Cor triatriatum: study of 20 cases. *The American Journal of Cardiology*.

[13] Lam C. R., Green E., Drake E. (1962). Diagnosis and surgical correction of 2 types of triatrial heart. *Surgery*.

[14] Lupinski R. W., Shankar S., Wong K. Y., Chan Y. H., Vosloo S. M., Moll J. J. (2001). Cor triatriatum: clinical presentation of 18 cases. *Asian Cardiovascular and Thoracic Annals*.

[15] Thilenius O. G., Bharati S., Lev M. (1976). Subdivided left atrium: an expanded concept of cor triatriatum sinistrum. *The American Journal of Cardiology*.

[16] Humpl T., Reineker K., Manlhiot C., Dipchand A. I., Coles J. G., McCrindle B. W. (2010). Cor triatriatum sinistrum in childhood. A single institution's experience. *Canadian Journal of Cardiology*.

[17] Wong C. K., Leung W. H., Cheng C. H., Lau C. P., Cheung D. L. C. (1989). Myxomatous mitral valve degeneration complicating asymptomatic cor triatriatum. *Clinical Cardiology*.

[18] Leung W.-H., Wong C.-K., Lau C.-P., Cheng C.-H. (1989). Cor triatriatum masked by coexisting COPD in an adult. *Chest*.

[19] Park K.-J., Park I.-K., Sir J.-J. (2009). Adult cor triatriatum presenting as cardioembolic stroke. *Internal Medicine*.

[20] Alabi F. O., Hernandez M., Christian F. G., Umeh F., Lama M. (2013). Pulmonary-esophageal variceal bleeding: a unique presentation of partial cor triatriatum sinistrum. *Case Reports in Vascular Medicine*.

[21] Chen Q., Guhathakurta S., Vadalapali G., Nalladaru Z., Easthope R. N., Sharma A. K. (1999). Cor triatriatum in adults: three new cases and a brief review. *Texas Heart Institute Journal*.

[22] Houston A., Hillis S., Lilley S., Richens T., Swan L. (1998). Echocardiography in adult congenital heart disease. *Heart*.

[23] Modi K. A., Annamali S., Ernest K., Pratep C. R. (2006). Diagnosis and surgical correction of cor triatriatum in an adult: combined use of transesophageal and contrast echocardiography, and a review of literature. *Echocardiography*.

[24] Bezgin T., Canga Y., Karagöz A. (2014). Multimodality imaging of cor triatriatum sinister in an octagenerian. *Echocardiography*.

[25] Einav E., Perk G., Kronzon I. (2008). Three-dimensional transthoracic echocardiographic evaluation of cor triatriatum. *European Journal of Echocardiography*.

[26] Ibrahim T., Schreiber K., Dennig K., Schömig A., Schwaiger M. (2003). Images in cardiovascular medicine. Assessment of cor triatriatum sinistrum by magnetic resonance imaging. *Circulation*.

[27] Tanaka F., Itoh M., Esaki H., Isobe J., Inoue R. (1991). Asymptomatic cor triatriatum incidentally revealed by computed tomography. *Chest*.

[28] Alphonso N., Nørgaard M. A., Newcomb A., D'Udekem Y., Brizard C. P., Cochrane A. (2005). Cor triatriatum: presentation, diagnosis and long-term surgical results. *The Annals of Thoracic Surgery*.

[29] Willens H. J., Ferrer P. L., Tamer D. F. (2010). Cor triatriatum sinister in an adult: management guided by real time three-dimensional transesophageal echocardiography and stress echocardiography. *Echocardiography*.

[30] Saxena P., Burkhart H. M., Schaff H. V., Daly R., Joyce L. D., Dearani J. A. (2014). Surgical repair of cor triatriatum sinister: the mayo clinic 50-year experience. *The Annals of Thoracic Surgery*.

[31] van Son J. A. M., Danielson G. K., Schaff H. V. (1993). Cor triatriatum: diagnosis, operative approach, and late results. *Mayo Clinic Proceedings*.

[32] Méndez A. B., Colchero T., Garcia-Picart J., Vila M., Subirana M. T., Sionis A. (2013). Unusual case of new-onset heart failure due to cor triatriatum sinister. *European Journal of Heart Failure*.

